# Genome sequence of the orange-pigmented seawater bacterium *Owenweeksia hongkongensis* type strain (UST20020801^T^)

**DOI:** 10.4056/sigs.3296896

**Published:** 2012-10-10

**Authors:** Thomas Riedel, Brittany Held, Matt Nolan, Susan Lucas, Alla Lapidus, Hope Tice, Tijana Glavina Del Rio, Jan-Fang Cheng, Cliff Han, Roxanne Tapia, Lynne A. Goodwin, Sam Pitluck, Konstantinos Liolios, Konstantinos Mavromatis, Ioanna Pagani, Natalia Ivanova, Natalia Mikhailova, Amrita Pati, Amy Chen, Krishna Palaniappan, Manfred Rohde, Brian J. Tindall, John C. Detter, Markus Göker, Tanja Woyke, James Bristow, Jonathan A. Eisen, Victor Markowitz, Philip Hugenholtz, Hans-Peter Klenk, Nikos C. Kyrpides

**Affiliations:** 1 HZI – Helmholtz Centre for Infection Research, Braunschweig, Germany; 2 DOE Joint Genome Institute, Walnut Creek, California, USA; 3 Los Alamos National Laboratory, Bioscience Division, Los Alamos, New Mexico, USA; 4 Biological Data Management and Technology Center, Lawrence Berkeley National Laboratory, Berkeley, California, USA; 5 Oak Ridge National Laboratory, Oak Ridge, Tennessee, USA; 6 Leibniz Institute DSMZ - German Collection of Microorganisms and Cell Cultures, Braunschweig, Germany; 7 University of California Davis Genome Center, Davis, California, USA; 8 Australian Centre for Ecogenomics, School of Chemistry and Molecular Biosciences, The University of Queensland, Brisbane, Australia

**Keywords:** aerobic, motile, rod-shaped, mesophilic, non-fermentative, Gram-negative, orange-pigmented sea water, *Bacteroidetes*, *Flavobacteria*, *Cryomorphaceae*, GEBA

## Abstract

*Owenweeksia hongkongensis* Lau *et al*. 2005 is the sole member of the monospecific genus *Owenweeksia* in the family *Cryomorphaceae,* a poorly characterized family at the genome level thus far. This family comprises seven genera within the class *Flavobacteria*. Family members are known to be psychrotolerant, rod-shaped and orange pigmented (β-carotene), typical for *Flavobacteria*. For growth, seawater and complex organic nutrients are necessary. The genome of *O. hongkongensis* UST20020801^T^ is only the second genome of a member of the family *Cryomorphaceae* whose sequence has been deciphered. Here we describe the features of this organism, together with the complete genome sequence and annotation. The 4,000,057 bp long chromosome with its 3,518 protein-coding and 45 RNA genes is a part of the *** G****enomic* ***E****ncyclopedia of* ***Bacteria**** and* ***Archaea***** project.

## Introduction

Strain UST20020801^T^ (= DSM 17368 = NRRL B-23963 = JCM 12287) is the type strain of the species *Owenweeksia hongkongensis* [1] in the monotypic genus *Owenweeksia* [1]. The genus was named after Owen B. Weeks for his work on *Flavobacterium* and *Cytophaga* during the 1950s to 1970s [1]. The species epithet points to Hong Kong, P. R. China, the place where the stain was isolated [1]. Strain UST20020801^T^ was first isolated in August 2002 from seawater samples collected from Port Shelter in Hong Kong during a study of the bacterial diversity in Hong Kong coastal sea water. Members of the phylum *Bacteroidetes* are widely distributed in marine and freshwater ecosystems. Compared to free-living bacteria, they were more frequently attached to aggregates [2,3] and occurred during algae blooms [4,5]. Representatives of the phylum *Bacteroidetes*, especially of the class *Flavobacteria*, are well-known to efficiently degrade and consume high-molecular-mass organic matter [6-11]. Recently, the family *Cryomorphaceae* was proposed to constitute a branch within the phylum *Bacteroidetes* [12]. This family encompasses the marine genera *Brumimicrobium*, *Cryomorpha*, *Crocinitomix* [12], *Owenweeksia* [1], *Lishizhenia* [13], *Wandonia* [14], and “*Phaeocystidibacter”* [15] as well as the freshwater-living genus *Fluviicola* [16]. Here we present a summary classification and a set of features for *O. hongkongensis* UST20020801^T^, together with the description of the genomic sequencing and annotation.

## Classification and features

A representative genomic 16S rRNA sequence of *O. hongkongensis* UST20020801^T^ was compared using NCBI BLAST [17,18] under default settings (e.g., considering only the high-scoring segment pairs (HSPs) from the best 250 hits) with the most recent release of the Greengenes database [19]. The relative frequencies of taxa and keywords (reduced to their stem [20]) were determined, weighted by BLAST scores. The only named genus in the list was *Owenweeksia* (1 hit in total). Regarding the single hit to a sequence from members of the species, the average identity within HSPs was 99.9%, whereas the average coverage by HSPs was 99.8%. No hits to sequences with other species names were found. (Note that the Greengenes database uses the INSDC (= EMBL/NCBI/DDBJ) annotation, which is not an authoritative source for nomenclature or classification.) The highest-scoring environmental sequence was EU328017 ('dynamics during bioremediation crude oil contaminated moderate saline soil clone B76'), which showed an identity of 93.2% and an HSP coverage of 99.9%. The most frequently occurring keywords within the labels of all environmental samples which yielded hits were 'marine' (3.0%), 'lake' (2.9%), 'depth' (2.7%), 'water' (2.6%) and 'zone' (2.5%) (249 hits in total) and corresponded with the habitat from which strain UST20020801^T^ was isolated.

**Figure 1:**
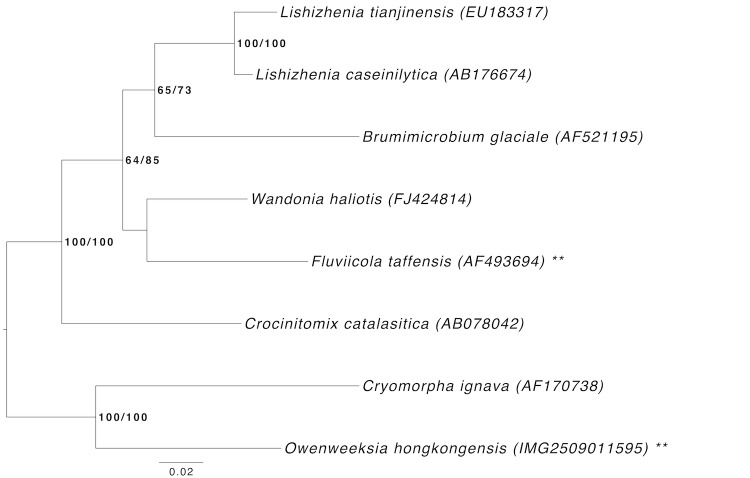
Phylogenetic tree highlighting the position of *O. hongkongensis* relative to the type strains of the other species within the family *Cryomorphaceae*. The tree was inferred from 1,409 aligned characters [21,22] of the 16S rRNA gene sequence under the maximum likelihood (ML) criterion [23]. Rooting was done initially using the midpoint method [24] and then checked for its agreement with the current classification (Table 1). The branches are scaled in terms of the expected number of substitutions per site. Numbers adjacent to the branches are support values from 400 ML bootstrap replicates [25] (left) and from 1,000 maximum-parsimony bootstrap replicates [26] (right) if larger than 60%. Lineages with type strain genome sequencing projects registered in GOLD [27] are labeled with one asterisk, those also listed as 'Complete and Published' with two asterisks [28].

Figure 1 shows the phylogenetic neighborhood of *O. hongkongensis* in a 16S rRNA based tree. The sequences of the two identical 16S rRNA gene copies in the genome do not differ from the previously published 16S rRNA sequence (AB125062).

**Table 1:** Classification and general features of *O. hongkongensis* UST20020801^T^ according to the MIGS recommendations [29] and NamesforLife [30].

**MIGS ID**	**Property**	**Term**	**Evidence code**
		Domain *Bacteria*	TAS [31]
		Phylum *Bacteroidetes*	TAs [32,33]
		Class *Flavobacteria*	TAS [34-36]
	Current classification	Order *Flavobacteriales*	TAS [33,37,38]
		Family *Cryomorphaceae*	TAS [12]
		Genus *Owenweeksia*	TAS [1]
		Species *Owenweeksia hongkongensis*	TAS [1]
		Strain UST20020801	TAS [1]
	Gram stain	negative	TAS [1]
	Cell shape	rod-shaped	TAS [1]
	Motility	motile	TAS [1]
	Sporulation	none	TAS [1]
	Temperature range	mesophile, 4-37°C	TAS [1]
	Optimum temperature	25-33°C	TAS [1]
	Salinity	1.0-7.5% NaCl (w/v), 0-100% sea water	TAS [1]
MIGS-22	Oxygen requirement	aerobe	TAS [1]
	Carbon source	yeast extract, peptone, starch	TAS [1]
	Energy metabolism	heterotroph	TAS [1]
MIGS-6	Habitat	Seawater	TAS [1]
MIGS-15	Biotic relationship	free-living	TAS [1]
MIGS-14	Pathogenicity	none	NAS
	Biosafety level	1	TAS [39]
MIGS-23.1	Isolation	sea water (sand-filtered)	TAS [1]
MIGS-4	Geographic location	Port Shelter, Hong Kong, China	TAS [1]
MIGS-5	Sample collection time	August 2002	TAS [1]
MIGS-4.1	Latitude	22.341	NAS
MIGS-4.2	Longitude	114.281	NAS
MIGS-4.3	Depth	5 m	TAS [1]
MIGS-4.4	Altitude	not reported	

Evidence codes - TAS: Traceable Author Statement (i.e., a direct report exists in the literature); NAS: Non-traceable Author Statement (i.e., not directly observed for the living, isolated sample, but based on a generally accepted property for the species, or anecdotal evidence). Evidence codes are from the Gene Ontology project [40].

**Figure 2:**
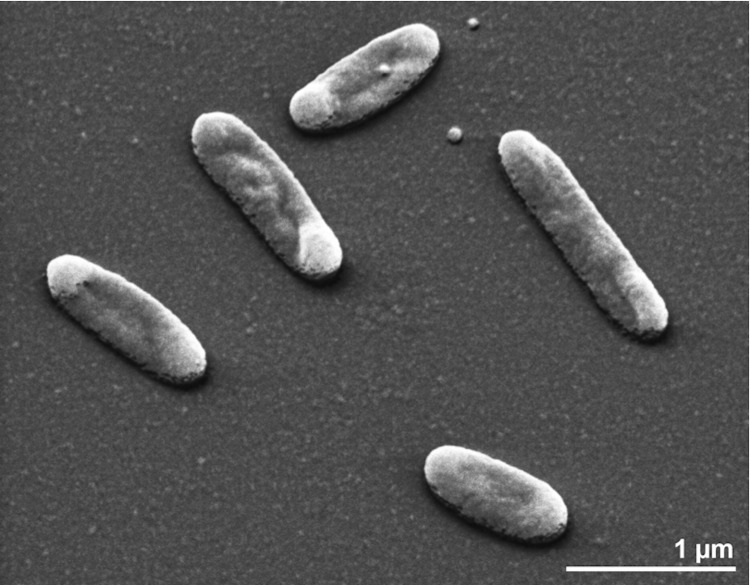
Scanning electron micrograph of *O. hongkongensis* UST20020801^T^

*O. hongkongensis* UST20020801^T^ is a Gram-negative, halophilic, non-flagellated, motile, and rod-shaped bacterium (Figure 2) [1]. Colonies are orange, convex, smooth, glistening and translucent with an entire margin [1]. Cells are 0.3-0.5 µm in width and 0.5-4.0 µm in length [1]. The strain does not sporulate [1]. Cells are strictly aerobic heterotrophs requiring Na^+^, Mg^2+^, sea salts and yeast extract or peptone for growth [1]. Growth occurs between 4°C and 37°C with an optimum at 25°C-33°C [1]. The pH range for growth is 5.2-9.0 with an optimum at pH 6.0-8.0 [1]. The salinity range for growth is 1.0-7.5% NaCl as well as 15-100% sea-water [1]. Yeast extract, peptone or starch is required for growth [1]. Ampicillin (10 µg), chloramphenicol (30 µg), erythromycin (10 µg), penicillin G (2U), rifampicin (10 µg), streptomycin (10 µg), tetracycline (30 µg) and polymyxin B (300 U) inhibited growth whereas cells were resistant to kanamycin (10 µg), gentamycin sulphate (10 µg) and spectinomycin (10 µg) [1]. Cells contain oxidase, catalase and alkaline phosphatase [1].

### Chemotaxonomy

The fatty-acid profile of strain UST20020801^T^ differs significantly from those of other members of the *Cryomorphaceae* [1]. The principal cellular fatty acids of strain UST20020801^T^ were the following saturated branched-chain fatty acids: *iso-*C_15:0 G_ (28.0%), *iso-*C_15:0_ (18.7%), *iso-*C_17:0 3-OH_ (18.1%), *iso-*C_17:1 ω9c_ (7.3%), *iso-*C_15:0 3-OH_ (4.9%), and a summed feature containing *iso*-C_15:0 2-OH_ and/or C_16:1ω7c_ (10.0%) [1]. Strain UST20020801^T^ had the highest level of *iso-*C_17:0 3-OH_ within *Cryomorphaceae*. Compared with other members of the *Cryomorphaceae*, the strain most similar to strain UST20020801^T^ with respect to the content of straight-chain fatty acids and branched-chain hydroxy fatty acids is *Cryomorpha ignava* 1-22^T^ [1]. In addition to phosphatidylethanolamine as major polar lipid, six unidentified lipids, one unidentified aminolipid, one unidentified aminophospholipid and one unidentified glycolipid were found in strain UST20020801^T^ [15]. MK-6 was detected as a major respiratory quinone in strain UST20020801^T^ [1].

## Genome sequencing and annotation

### Genome project history

This organism was selected for sequencing on the basis of its phylogenetic position [41], and is part of the *** G****enomic* ***E****ncyclopedia of* ***Bacteria**** and* ***Archaea***** project [42]. The genome project is deposited in the Genomes On Line Database [27] and the complete genome sequence is deposited in GenBank. Sequencing, finishing and annotation were performed by the DOE Joint Genome Institute (JGI). A summary of the project information is shown in Table 2.

**Table 2:** Genome sequencing project information

**MIGS ID**	**Property**	**Term**
MIGS-31	Finishing quality	Finished
MIGS-28	Libraries used	Three genomic libraries: one 454 pyrosequence standard library, one 454 PE library (8.5 kb insert size), one Illumina library
MIGS-29	Sequencing platforms	Illumina GAii, 454 GS FLX Titanium
MIGS-31.2	Sequencing coverage	300.0 × Illumina; 8.6 × pyrosequence
MIGS-30	Assemblers	Newbler version 2.3-PreRelease-6/30/2009, Velvet 1.0.13, phrap version SPS - 4.24
MIGS-32	Gene calling method	Prodigal
	INSDC ID	CP003156
	GenBank Date of Release	June 15, 2012
	GOLD ID	Gc02043
	NCBI project ID	65297
	Database: IMG-GEBA	2508501098
MIGS-13	Source material identifier	DSM 17368
	Project relevance	Tree of Life, GEBA

### Growth conditions and DNA isolation

*O. hongkongensis* strain UST20020801^T^, DSM 17368, was grown in DSMZ medium 1168 (YPS medium) [43] at 30°C. DNA was isolated from 0.5-1 g of cell paste using Jetflex Genomic DNA Purification kit (GENOMED 600100) following the standard protocol as recommended by the manufacturer with an extended cell-lysis procedure, i.e. incubation with additional 80 µl protease K for one hour at 58°C. DNA is available through the DNA Bank Network [44].

### Genome sequencing and assembly

The genome was sequenced using a combination of Illumina and 454 sequencing platforms. All general aspects of library construction and sequencing can be found at the JGI website [45]. Pyrosequencing reads were assembled using the Newbler assembler (Roche). The initial Newbler assembly, consisting of 39 contigs in one scaffold, was converted into a phrap [46] assembly by making fake reads from the consensus to collect the read pairs in the 454 paired end library. Illumina GAii sequencing data (5,738.3 Mb) was assembled with Velvet [47] and the consensus sequences were shredded into 1.5 kb overlapped fake reads and assembled together with the 454 data. The 454 draft assembly was based on 81.1 Mb 454 draft data and all of the 454 paired end data. Newbler parameters are -consed -a 50 -l 350 -g -m -ml 20. The Phred/Phrap/Consed software package [46] was used for sequence assembly and quality assessment in the subsequent finishing process. After the shotgun stage, reads were assembled with parallel phrap (High Performance Software, LLC). Possible mis-assemblies were corrected with gapResolution [45], Dupfinisher [48], or sequencing cloned bridging PCR fragments with subcloning. Gaps between contigs were closed by editing in Consed, by PCR and by Bubble PCR primer walks (J.-F. Chang, unpublished). A total of 58 additional reactions were necessary to close gaps and to raise the quality of the finished sequence. Illumina reads were also used to correct potential base errors and increase consensus quality using the software Polisher developed at JGI [49]. The error rate of the completed genome sequence is less than 1 in 100,000. Together, the combination of the Illumina and 454 sequencing platforms provided 308.6 x coverage of the genome. The final assembly contained 291,505 pyrosequence and 75,503,620 Illumina reads.

### Genome annotation

Genes were identified using Prodigal [50] as part of the Oak Ridge National Laboratory genome-annotation pipeline, followed by a round of manual curation using the JGI GenePRIMP pipeline [51]. The predicted CDSs were translated and used to search the National Center for Biotechnology Information (NCBI) non-redundant database, UniProt, TIGRFam, Pfam, PRIAM, KEGG, COG, and InterPro databases. These data sources were combined to assert a product description for each predicted protein. Additional gene prediction analysis and functional annotation was performed within the Integrated Microbial Genomes - Expert Review (IMG-ER) platform [52].

## Genome properties

**Figure 3:**
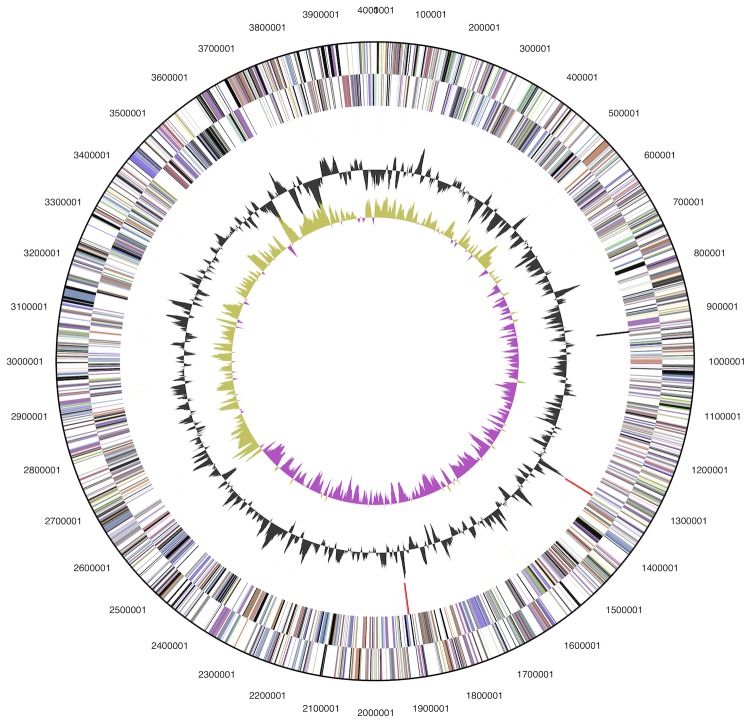
Graphical map of the chromosome. From outside to center: Genes on forward strand (colored by COG categories), Genes on reverse strand (colored by COG categories), RNA genes (tRNAs green, rRNAs red, other RNAs black), GC content (black), GC skew (purple/olive).

The genome consists of a 4,000,057 bp long circular chromosome with a G+C content of 40.2% (Figure 3 and Table 3). Of the 3,563 genes predicted, 3,518 were protein-coding genes, and 45 RNAs; 33 pseudogenes were also identified. The majority of the protein-coding genes (67.9%) were assigned a putative function while the remaining ones were annotated as hypothetical proteins. The distribution of genes into COGs functional categories is presented in Table 4.

**Table 3:** Genome Statistics

**Attribute**	**Value**	**% of Total**
Genome size (bp)	4,000,057	100.00%
DNA coding region (bp)	3,661,831	91.54%
DNA G+C content (bp)	1,609,363	40.23%
Number of replicons	1	
Extrachromosomal elements	0	
Total genes	3,563	100.00%
RNA genes	45	1.26%
rRNA operons	2	
tRNA genes	38	0.93%
Protein-coding genes	3,518	98.74%
Pseudo genes	33	0.93%
Genes with function prediction (proteins)	2,301	64.58%
Genes in paralog clusters	1,516	42.55%
Genes assigned to COGs	2,279	63.96%
Genes assigned Pfam domains	2,263	66.32%
Genes with signal peptides	1,095	30.73%
Genes with transmembrane helices	822	23.07%
CRISPR repeats	0	

**Table 4:** Number of genes associated with the general COG functional categories

**Code**	**Value**	**%age**	**Description**
J	155	6.2	Translation, ribosomal structure and biogenesis
A	0	0.0	RNA processing and modification
K	139	5.6	Transcription
L	141	5.7	Replication, recombination and repair
B	1	0.0	Chromatin structure and dynamics
D	33	1.3	Cell cycle control, cell division, chromosome partitioning
Y	0	0.0	Nuclear structure
V	54	2.2	Defense mechanisms
T	147	5.9	Signal transduction mechanisms
M	233	9.3	Cell wall/membrane biogenesis
N	13	0.5	Cell motility
Z	3	0.1	Cytoskeleton
W	0	0.0	Extracellular structures
U	45	1.8	Intracellular trafficking and secretion, and vesicular transport
O	107	4.3	Posttranslational modification, protein turnover, chaperones
C	116	4.7	Energy production and conversion
G	71	2.9	Carbohydrate transport and metabolism
E	181	7.3	Amino acid transport and metabolism
F	57	2.3	Nucleotide transport and metabolism
H	115	4.6	Coenzyme transport and metabolism
I	114	4.6	Lipid transport and metabolism
P	127	5.1	Inorganic ion transport and metabolism
Q	54	2.2	Secondary metabolites biosynthesis, transport and catabolism
R	337	13.5	General function prediction only
S	251	10.1	Function unknown
-	1,284	36.0	Not in COGs

## Insights into the genome sequence

Genome analysis of strain UST20020801^T^ revealed the presence of genes encoding an arylsulfatase A family protein (Oweho_0043), a bacteriophytochrome (light-regulated signal transduction histidine kinase (Oweho_0350), a cytochrome c2 and a cytochrome c oxidase cbb3 type (Oweho_2085)). Additional gene sequences of interest encode a homogenisate 1,2-dioxigenase (Oweho_2010), a haloacid dehalogenase superfamily protein (Oweho_2094) as well as a 2-haloalkanoic acid dehalogenase type II (Oweho_2503). The presence of these genes could indicate that strain UST20020801^T^ plays a role in the respiratory degradation of recalcitrant compounds in its ecological niche. Further, a light-dependent regulation of metabolic activities using bacteriophytochrome as a sensor seems to be possible.
